# Impact of Living Environment on 2-Year Mortality in Elderly Maintenance Hemodialysis Patients

**DOI:** 10.1371/journal.pone.0074358

**Published:** 2013-09-18

**Authors:** Wen-Hung Huang, Ja-Liang Lin, Dan-Tzu Lin-Tan, Kuan-Hsing Chen, Ching-Wei Hsu, Tzung-Hai Yen

**Affiliations:** Division of Nephrology and Clinical Toxicology, Chang Gung Memorial Hospital, Lin-Kou Medical Center, Chang Gung University and School of Medicine, Taipei, Taiwan, ROC; National University of Singapore, Singapore

## Abstract

**Background:**

Studies on risk factors of mortality in elderly patients with hemodialysis usually focus on comorbidities, nutrition, and inflammation. Discussion on the correlation between living environment and mortality of these patients is limited.

**Methods:**

A total of 256 elderly hemodialysis patients participated in this 2-year prospective observational study. The subjects were divided into 2 subgroups based on whether they were living in Taipei Basin (n = 63) or not (n = 193). Demographic, hematological, nutritional, inflammatory, biochemical, and dialysis-related data were obtained for cross-sectional analysis. Causes of death and mortality rates were also analyzed for each subgroup.

**Results:**

Patients in the basin group had a higher incidence of combined protein-energy wasting and inflammation than those in the around basin group. At the end of the 2-year follow-up, 68 patients had died. Univariate binary logistic regression analysis revealed that a very advanced age, basin group, serum albumin levels, serum creatinine levels, non-anuria, and the complications of stroke and CAD were associated with 2-year mortality. Meanwhile, log high-sensitivity C-reactive protein (hs-CRP) levels were not associated with 2-year mortality. Multivariate Cox regression analysis revealed that basin group, serum albumin levels, and the complications of stroke and CAD were significant risk factors for 2-year mortality in these patients.

**Conclusion:**

The results of this study indicate that factors such as living in the Taipei Basin with higher air pollutant levels in elderly hemodialysis patients is associated with protein-energy wasting and inflammation, as well as 2-year mortality. These findings suggest that among this population, living environment is as important as comorbidities and nutrition. Furthermore, air pollution should be getting more attention especially in the overcrowding Basin topography.

## Introduction

The prevalence of dialysis in elderly patients is increasing worldwide [1], owing to advanced health insurance [2] and better management of comorbidities and chronic diseases such as diabetes mellitus (DM), cardiovascular disease, cerebral vascular disease, and malignancy. Dialysis in the elderly is complicated. Age-related problems are associated with increased risk factors in elderly patients with end-stage renal disease (ESRD) [3], [4]. However, when survival is compared within older populations, the risk of age is controversial [5]–[7]. Short-term survival for elderly patients starting dialysis varies from 50% to 80% [1], [2]. In a study on short-term survival of elderly patients on dialysis, increased alcohol consumption, cardiac dyskinesis, age at onset of dialysis, serum phosphate, and number of comorbid illnesses were correlated with the risk of mortality and normal serum albumin reduced the risk of mortality [7]. Therefore, dialysis should be given careful consideration in elderly patients. Functional dependence, impaired intellectual status, diabetes, low serum albumin, peripheral vascular disease, and late referral for ESRD treatment are also poor prognostic factors in this population [5]. In patients with these comorbidities, and especially in those with coronary heart disease, dialysis might not offer a survival advantage [8], [9]. To the best of our knowledge, studies on elderly hemodialysis (HD) patients mostly focus on nutrition and comorbidities, and study on living environment is limited. The aim of this prospective observational study was to assess the effect of living area and other important mortality-associated factors on 2-year mortality in elderly HD patients.

## Methods

This is a prospective observational study of elderly patients with receiving routinely hemodialysis in three hemodialysis centers (Hemodialysis centers of Chang Gung Memorial Hospital in Taipei, Lin-Kou Medical Center and Taoyuan branch). Recruitment started in January 2009 and ended in March 2009. Follow up ended in April 2011. All individuals aged ≥65 years consenting to participate and responding to informed consent were included. This study complied with the guidelines of the Declaration of Helsinki and approved by the Medical Ethics Committee of Chang Gung Memorial Hospital, a tertiary referral center located in the northern part of Taiwan. Written informed consent was obtained from every participating subject, and the study was approved by the institutional review board of the Chang Gung Memorial Hospital. In addition, all individual information was securely protected (by delinking identifying information from main data set) and available to investigators only. Furthermore, all the data were analyzed anonymously. Finally, all primary data were collected according to strengthening the reporting of observational studies in epidemiology guidelines.

### Patients

All patients studied were aged 65 years or older, were recruited from 3 HD centers at our hospital, and gave informed consent to participate in this study. Subjects with malignancies and active infectious diseases were excluded, as were those who had received regular HD for <6 months, or been hospitalized or undergone surgery or renal transplantation within 3 months before the study. Most HD patients were undergoing 4 h of HD 3 times per week. The patients were treated using single-use hollow-fiber dialyzers fitted with modified cellulose-based polyamide or polysulfone membranes. The dialysate was a standard ionic composition bicarbonate-based buffer, and a standard reverse osmosis system was used for water purification. Complications such as cardiomegaly, gastrointestinal bleeding, hepatitis, cirrhosis, stroke, asthma, pulmonary tuberculosis (Pul TB), pleural effusion, neuropathy, carpal tunnel syndrome, skin lesion, cancer, and coronary artery disease (CAD) were assessed by studying the patients’ medical records. Non-anuria status was defined as a daily urine volume greater than 100 mL. Very advanced age was defined as an age over 75 years.

**Figure 1:**
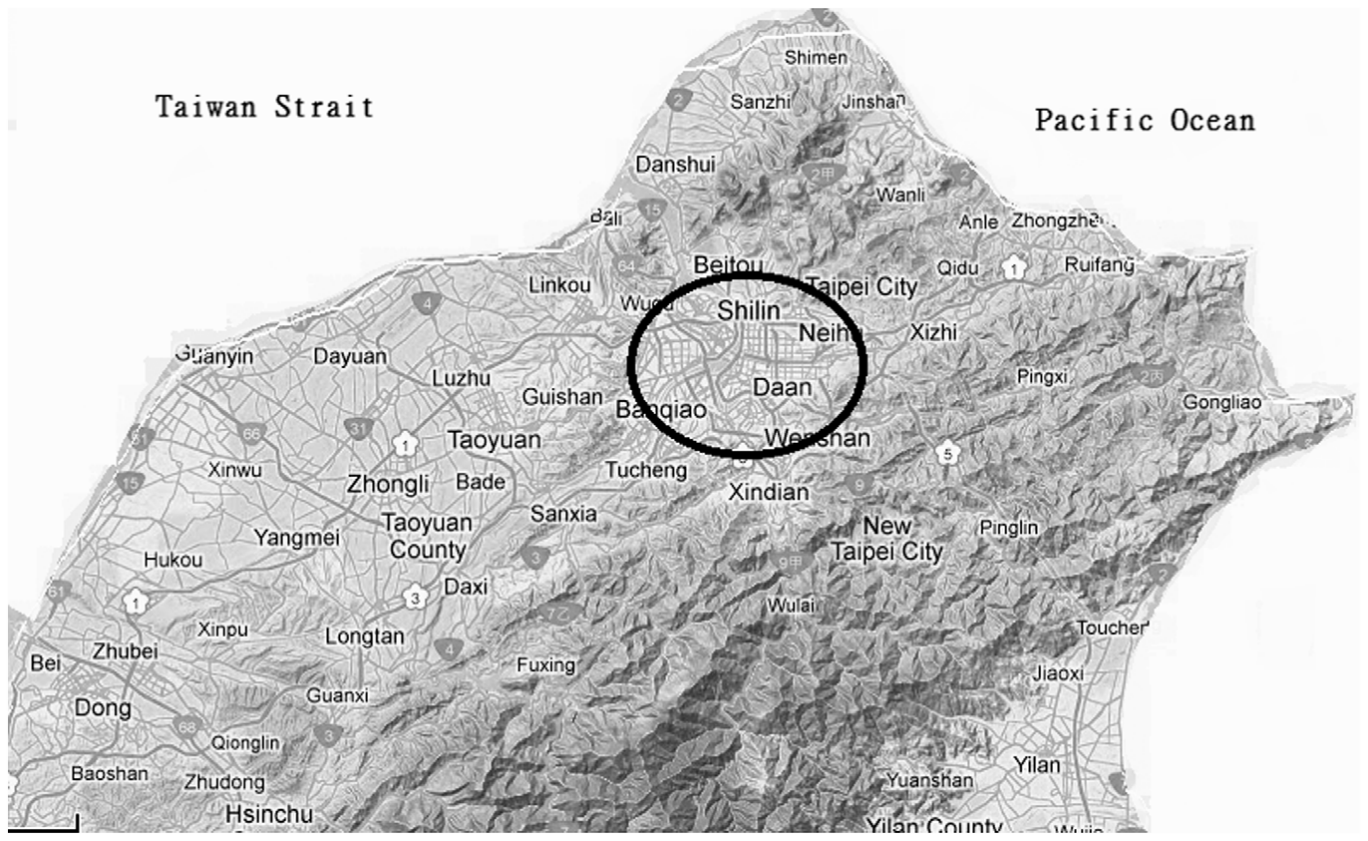
Terrain of northern Taiwan: The Taipei Basin (circle part, including most of Taipei City) which is surrounded by mountains and hills has 6 million people living in it. The map was cited from Google map with adjustment.

Living areas were divided into in Taipei Basin and around Taipei Basin. The coverage of Taipei Basin include: East, Nankang District of Taipei City; West, Shulin District of New Taipei City; South, Jingmei District of Taipei City; North, Beitou District of Taipei City (figure 1).

### Laboratory, Nutritional, and Inflammatory Parameters

Laboratory data for each subject were obtained during stable outpatient HD sessions to minimize the influence of acute events at the beginning of the study. Blood samples were taken from the arterial end of the vascular access immediately before initiating the mid-week HD session; samples were then centrifuged and stored at −70°C until assay. Serum albumin, creatinine levels, and normalized protein catabolism rate (nPCR) were assayed and recorded as nutritional markers. White blood cell count, platelets, and hemoglobin were measured as hematological indicators. Both serum ferritin and high-sensitivity C-reactive protein (hs-CRP) were used as inflammatory markers. Serum hs-CRP concentrations were measured using immunonephelometry (Nanopia CRP; Daiichi, Tokyo, Japan), with a minimum detection limit of 0.15 mg/L. All other markers were measured by an automatic analyzer by using standard laboratory procedures. The nPCR was calculated via validated equations and normalized to actual body weight [10]. Dialysis clearance of urea was expressed as Kt/V_urea_, by using the method described by Daugirdas [11] in HD patients. Serum calcium, phosphate, and intact parathyroid hormone (i-PTH) were also measured. Corrected calcium (C-calcium) concentration was calculated using the following equation: C-calcium = serum calcium (mg/dL)+[0.8 (4.0 − serum albumin (g/dL))].

### Definitions of Protein-energy Wasting and Inflammation

To determine whether urban life was associated with the inflammation and/or protein-energy wasting status of maintenance HD patients, this study investigated serum albumin and hs-CRP levels in different subgroups. Due to the lack of any definite hs-CRP cutoff level indicating an inflammatory state in maintenance HD patients, inflammation was defined as an hs-CRP level >3 mg/L, a level correlating with increased cardiovascular risk in the general population [12]. This study defined protein-energy wasting as a serum albumin level <3.6 g/dL (<36 g/L), a level that represents the 10th percentile of the Third National Health and Nutrition Examination Survey [13],[14]. Using the above criteria, we divided the patients into 3 groups: group 1, normal; group 2, inflammation or protein-energy wasting; and group 3, combined protein-energy wasting and inflammation.

### Air Quality Status and Analysis

**Figure 2:**
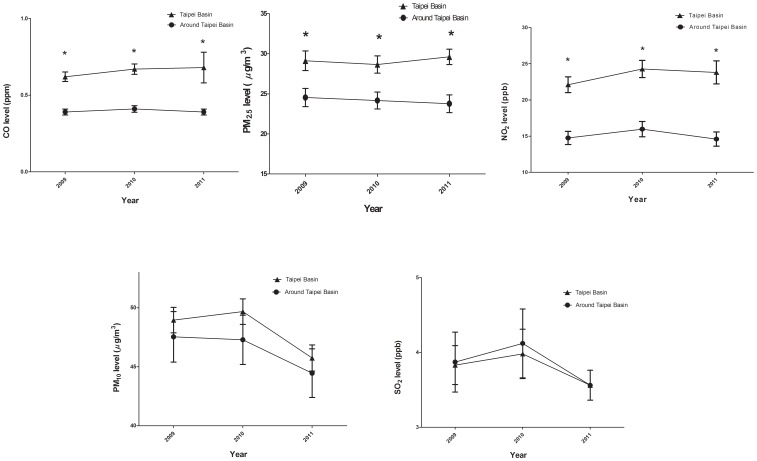
Analyzed the data base from the Taiwan Air Quality Monitoring Network (TAQMN) operated by the Environmental Protection Administration (EPA)and cited the report from the air quality status in Taiwan for the year 2009 to 2011. The mean concentrations of PM_2.5_, NO_2_ and CO were significantly high in Taipei Basin during these 3 years. *, significant difference, *P*<0.05.

In order to verify our inference, we analyzed the data base and cited the report from the air quality status in Taiwan for the year 2009 to year 2011 based on the data from the Taiwan Air Quality Monitoring Network (TAQMN) operated by the Environmental Protection Administration (EPA) [15]. We analyzed the air quality difference between the Taipei Basin and around the Taipei Basin. The referenced items including 1year average concentration of particulate matter with aerodynamic diameter less than 10 and 2.5 µm (PM_10_ and PM_2.5_), sulfur dioxide (SO_2_), nitrogen dioxide (NO_2_) and carbon monoxide (CO). Air pollution levels were recorded by a network of 14 and 10 monitoring stations spreading in Taipei Basin and around Taipei Basin, respectively. Comparing above items between two areas from year 2009 to year 2011, the mean concentrations of PM_2.5_, NO_2_ and CO were significantly high in Taipei Basin (figure 2) and that of SO_2_ and PM_10_ were not significantly different.

### Statistical Analysis

The data are expressed as median and interquartile range for non-normally distributed variables and as mean ± SD for normally distributed variables. Comparisons between groups were performed using the Mann–Whitney test and Student’s t test. Chi-square or Fisher’s exact tests were used to analyze the correlation between categorical variables. Mortality data were compared using the Kaplan–Meier method and significance was tested using a log-rank test. Logarithmic conversion was made for i-PTH and hs-CRP levels. Initially, univariate logistic regression analysis was used to identify the biochemical and demographic variables correlated with 2-year mortality. The following factors were investigated: very advanced age, gender, body mass index (weight/height^2^), HD years, smoking status, cardiovascular disease, viral hepatitis B and C, hypertension, urban life, use of arteriovenous (AV) fistula, tunneled-cuffed catheter (TCC), AV graft for vascular access, biocompatible membrane dialyzers, calcium, phosphate, i-PTH, and Kt/V_urea_ (Daugirdas). Multivariate Cox regression analysis (stepwise forward approach) was then applied to assess 2-year mortality predictors. To calculate the relative risk of death, hazard ratios (HRs) and 95% confidence intervals (95% CIs) were obtained by Cox regression analysis. The possible independent variables (from the significant variables in univariate logistic regression) included very advanced age, gender, urban life, serum albumin levels, serum creatinine levels, a status of non-anuria, and the complications of stroke and CAD. Statistical significance was set at p<0.05. All statistical analyses were performed using the Statistical Package for the Social Sciences (SPSS) Version 12.0 for Windows (SPSS Inc., Chicago, IL, USA).

## Results

Initially, 271 elderly patients were enrolled in this study. Fifteen subjects were excluded because of missing data. Finally, 256 patients (142 women and 114 men) were recruited. Table 1 lists the baseline clinical characteristics of the patients, including age, gender, body mass index, and biological and hematological data. The mean age of the patients was 72.6±5.6 years. Among the patients, 80 had a medical history of DM, 39 were habitual users of tobacco, 20 had hepatitis B virus infection, and 40 had hepatitis C virus infection. Sixty-three patients lived in Taipei Basin, Taiwan, (basin group) and the other 193 patients were not living around Taipei Basin (around basin group). With regards to vascular access, 173 patients were using AV fistula, 65 patients were using AV graft, and 18 patients were using TCC. Primary renal diseases present included chronic glomerulonephritis in 51 patients (19.9%) and hypertension-related nephropathy in 101 patients (39.4%). Ninety-three patients (36.3%) were older than the age of 75 years.

**Table 1:** Baseline characteristics of the patients studied (n = 256).

Characteristics	
**Demographics**	
**Age, years**	72.64±5.64
**Very advanced age, no. of patients**	93
**Body mass index, kg/m^2^**	22.13±3.25
**Male gender, no. of patients**	114
**Smoking, no. of patients**	39
**Previous DM, no. of patients**	80
**Previous cardiovascular diseases, no. of patients**	15
**Hypertension, no. of patients**	101
**Chronic hepatitis B, no. of patients**	20
**Chronic hepatitis C, no. of patients**	40
**Living in Taipei Basin, no. of patients**	63
**Dialysis-related data**	
**Hemodialysis duration, years**	6.02±4.64
**Kt/V_urea_ (Daugirdas)**	1.81±0.31
**nPCR, g•kg^−1^•day^−1^**	1.1±0.26
**Biochemical data**	
**White blood cell count, ×10^3^/µL**	6.4±1.99
**Hemoglobin, g/dL**	10.2±1.33
**Platelets, ×10^3^/µL**	185.25±60.52
**Albumin, g/dL**	3.9±0.34
**Creatinine, mg/dL**	9.6±2.07
**Transferrin saturation, %**	30.98±12.64
**Ferritin, µg/L**	458.47±442.3
**C-Calcium, mg/dL**	9.87±0.94
**Phosphate, mg/dL**	4.27±1.25
**Intact parathyroid hormone, pg/mL**	96.7 [34.2–235.4]
**Cardiovascular risk factors**	
**Cholesterol, mg/dL**	166.44±38.89
**HDL-C, mg/dL**	42.94±13.77
**LDL-C, mg/dL**	92.86±31.17
**Triglyceride, mg/dL**	158.9±122.75
**Hs-CRP, mg/L**	4.74 [2.1–9.8]
**Cardiothoracic ratio, %**	52.9±7.06

*Abbreviations:* DM, diabetes mellitus; AV, arteriovenous; TCC, tunneled-cuffed catheter; HDF, hemodiafiltration; nPCR, normalized protein catabolism rate; C-calcium, corrected calcium; HDL-C, high-density lipoprotein cholesterol; LDL-C, low-density lipoprotein cholesterol; Hs-CRP, high-sensitivity C-reactive protein.

**Figure 3:**
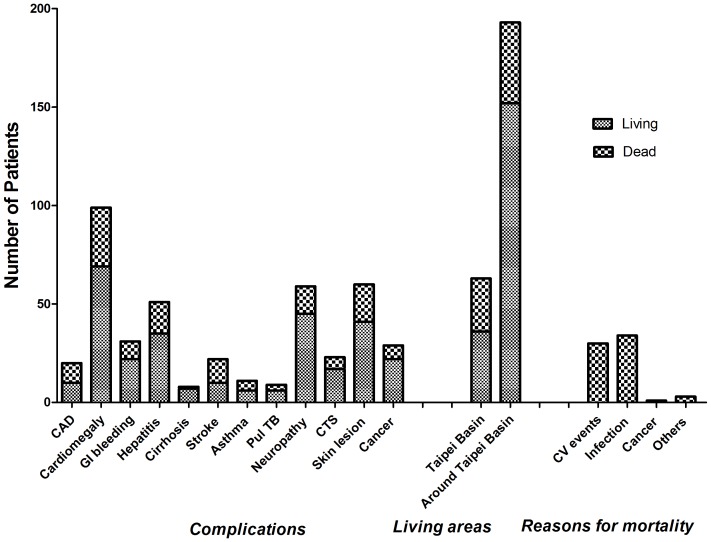
Complications, living areas, and reasons for mortality in 256 elderly patients. Sixty-eight patients (26.5%) died within 2 years. Infection (50%) and cardiovascular events (44.1%) were the major causes of 2-year mortality. Complications such as stroke (OR: 3.814, 95% CI (1.56–9.30); p = 0.004) and coronary artery disease (OR: 3.069, 95% CI (1.21–7.74); p = 0.019) were positively associated with mortality. Cancer status, asthma, pulmonary tuberculosis, hepatitis, liver cirrhosis, and cardiomegaly were not significantly associated with 2-year mortality. *Abbreviations:* CAD, coronary artery disease; GI, gastrointestinal; Pul TB, pulmonary tuberculosis; CTS, carpal tunnel syndrome; CV, cardiovascular.

Sixty-eight patients (26.5%) died within 2 years. Infection (50%) and cardiovascular events (44.1%) were the main causes of 2-year mortality. Complications such as stroke (OR: 3.814, 95% CI (1.56–9.30); p = 0.004) and CAD (OR: 3.069, 95% CI (1.21–7.74); p = 0.019) were positively associated with mortality. Cancer status, asthma, Pul TB, hepatitis, liver cirrhosis, and cardiomegaly were not significantly associated with 2-year mortality (Figure 3). To further investigate the influence of clinical features on 2-year mortality, we used a univariate binary logistic regression analysis to evaluate the association between mortality and clinical variables in the patients. A very advanced age (OR: 1.851, 95% CI (1.05–3.25); p = 0.033), basin group (OR: 2.78, 95% CI (1.51–5.1); p = 0.001), serum albumin levels (OR: 0.192, 95% CI (0.081–0.458); p<0.001), serum creatinine levels (OR: 0.851, 95% CI (0.742–0.977); p = 0.022), non-anuria (OR: 0.395, 95% CI (0.159–0.983); p = 0.046), and the complications of stroke (OR: 3.814, 95% CI (1.56–9.30); p = 0.003) and CAD (OR: 3.06, 95% CI (1.21–7.74); p = 0.018) were associated with 2-year mortality. Meanwhile, log hs-CRP levels were not associated with 2-year mortality (OR: 1.57, 95% CI (0.86–2.87); p = 0.137). Further, multivariate Cox regression analysis revealed that basin group (HR: 1.762, 95% CI (1.054–2.947); p = 0.031), serum albumin levels (HR: 0.304, 95% CI (0.157–0.588); p<0.001), and the complications of stroke (HR: 3.124, 95% CI (1.639–5.955); p = 0.001) and CAD (HR: 2.031, 95% CI (1.008–4.094); p = 0.048) were significant risk factors for 2-year mortality (Table 2).

**Table 2:** Binary univariate logistic regression and multivariate Cox regression analysis of variables on 2-year mortality.

Variable	Univariate binary logisticregression analysis OR (95% CI)	p value	Multivariate Cox regressionanalysis HR (95% CI)	p value
**Very advanced age (>75 years)**	1.851 (1.05–3.25)	0.033		
**Male gender**	0.901 (0.51–1.57)	0.715		
**Basin group**	2.78 (1.51–5.1)	0.001	1.762 (1.054–2.947)	0.031
**Serum Cr level**	0.851 (0.742–0.977)	0.022		
**Non-anuria**	0.395 (0.159–0.983)	0.046		
**Albumin, g/dL (each increment of 1 g/dL)**	0.192 (0.081–0.458)	<0.001	0.304 (0.157–0.588)	<0.001
**Stroke**	3.814 (1.56–9.30)	0.003	3.124 (1.639–5.955)	0.001
**CAD**	3.06 (1.21–7.74)	0.018	2.031 (1.008–4.094)	0.048
**Log hs-CRP**	1.577 (0.86–2.87)	0.137		

*Abbreviations:* Cr, creatinine; CAD, coronary artery disease; log hs-CRP, logarithmic conversion of high-sensitivity C-reactive protein levels; OR, odds ratio; HR, hazard ratio; CI, confidence intervals.

**Figure 4:**
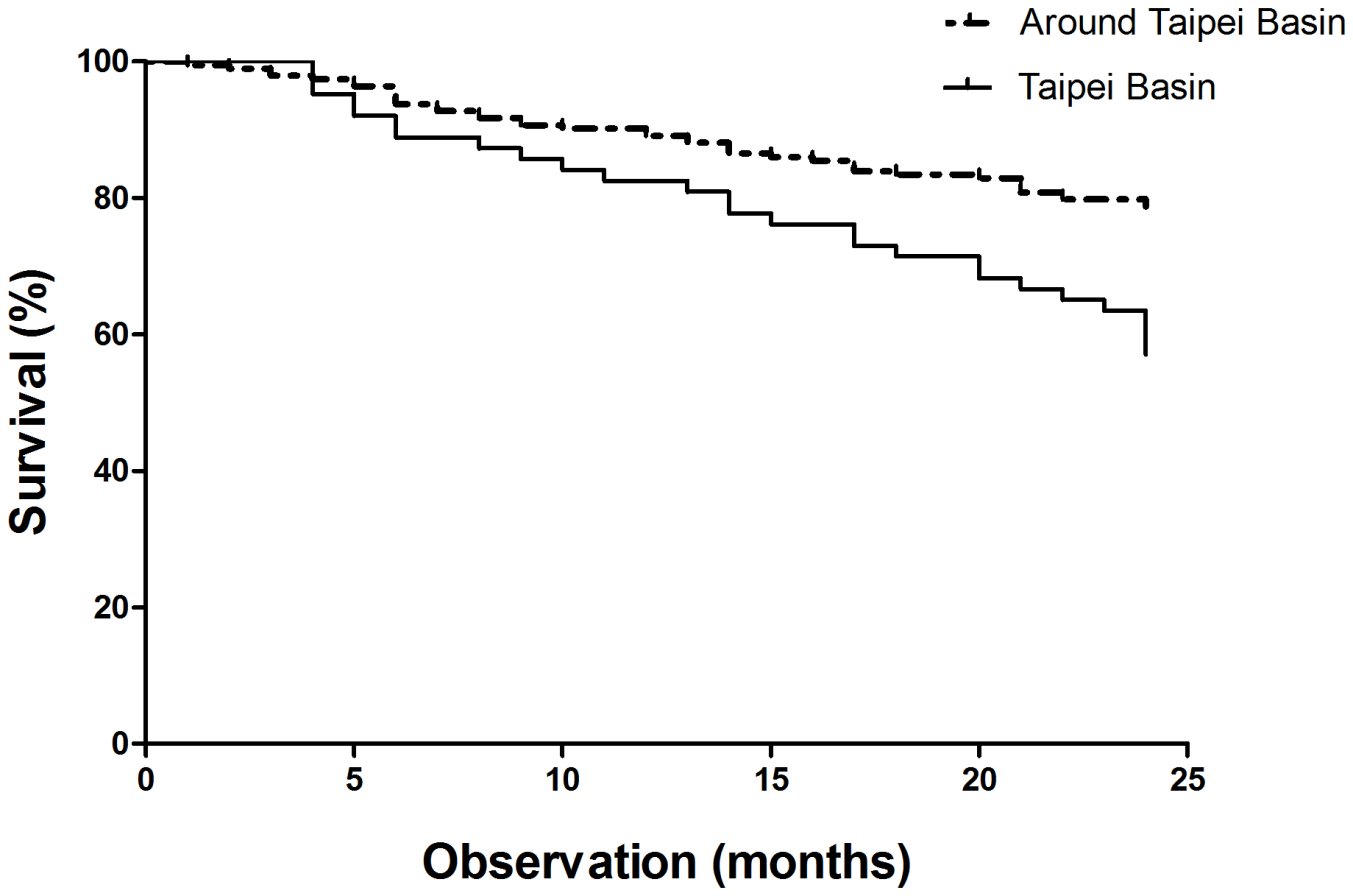
Kaplan–Meier analysis of survival data from patients living in Taipei Basin and those living around Taipei Basin. Patients living in Taipei Basin suffered higher cumulative mortality than patients living areas around Taipei Basin (log-rank test, chi-square test = 11.01; p = 0.001).

In order to clarify the association between basin group and around basin group, subgroup analysis was used for patients living in these two areas. The basin group had a lower nPCR than the around basin group (1.04±0.21 vs. 1.12±0.27 g⋅kg^−1^⋅day^−1^; p = 0.027) (Table 3). Age (73.6±5.6 vs. 72.3±5.6 years), body mass index (22.19±3.1 vs. 21.96±3.71 kg/m^2^), hemodialysis duration (5.41±3.83 vs. 6.53±4.86 years), serum albumin levels (3.90±0.35 vs. 3.91±0.34 g/dL), and log hs-CRP levels (0.75±0.45 vs. 0.63±0.48) were not significantly different (p>0.05) between patients living in Taipei Basin and living around Taipei Basin (Table 3). With regards to nutritional and inflammatory status in these two groups, we separated the patients into 3 groups: group 1, normal; group 2, protein-energy wasting or inflammation; group 3, combined protein-energy wasting and inflammation. The chi-square test revealed that the majority of patients in the combined protein-energy wasting and inflammation group belonged to the Basin group (19% vs. 7.8%; p = 0.041) (Table 4). Finally, Kaplan–Meier analysis showed that cumulative mortality was higher among Basin group than among around Basin group (log-rank test, chi-square test = 11.01; p = 0.001) (Figure 4). Moreover, the rate of the complications of stroke (9.5% vs. 8.3%; p>0.05) and CAD (12.7% vs. 6.3%; p>0.05) were not different between these 2 groups. Patients living in Taipei Basin had higher 2-year cardiovascular mortality than the around basin group (OR: 2.288, 95% CI (1.03–5.06); p = 0.044). However, the Basin group did not have a higher rate for 2-year infection mortality than the around Basin group (OR: 2.13, 95% CI (0.996–4.553); p = 0.056) (Table 5).

**Table 3:** Comparison of clinical variables between patients living in Taipei Basin and living around Taipei Basin.

	Around basin group (n = 193)	Basin group (n = 63)	p value
**Age, years**	72.3±5.6	73.6±5.6	0.109
**Body mass index, kg/m^2^**	22.19±3.1	21.96±3.71	0.672
**Female/Male**	109/84	33/30	0.662
**Hemodialysis duration, years**	6.53±4.86	5.41±3.83	0.063
**Anuria, no. of patients**	156	57	0.083
***Dialysis-related data***			
**Kt/V_urea_ (Daugirdas)**	1.82±0.31	1.76±0.27	0.142
**nPCR, g⋅kg^−1^⋅day^−1^**	1.12±0.27	1.04±0.21	0.027
**TAC_urea_, mg/100 mL**	37.68±10.76	35.63±8.89	0.133
**UF volume, L**	2.10±0.87	2.15±0.97	0.677
***Biochemical data***			
**Hemoglobin, g/dL**	10.13±1.4	10.43±1.2	0.1
**Albumin, g/dL**	3.91±0.34	3.90±0.35	0.893
**Creatinine, mg/dL**	9.51±2.13	9.88±1.89	0.193
**Ferritin, µg/L**	473.35±494.50	420.76±289.78	0.307
**C-Calcium, mg/dL**	9.9±0.99	9.8±0.83	0.537
**Phosphate, mg/dL**	4.36±1.22	4.09±1.28	0.143
**C-calcium×phosphate, mg^2^/dL^2^**	43.5±14.04	40.3±13.6	0.117
**ALT, U/L**	16.87±14.60	15.15±9.88	0.295
**Glucose, g/dL**	132.49±68.16	133.74±60.82	0.891
**Uric acid, mg/dL**	7.09±1.40	7.06±1.41	0.881
**Na, mEq/L**	139.52±3.01	139.14±3.08	0.381
**K, mEq/L**	4.84±0.70	4.65±0.65	0.052
**HbA1c, %**	7.43±1.98	7.06±1.59	0.412
***Cardiovascular risk factors***			
**Cholesterol, mg/dL**	165.64±40.63	167.26±32.83	0.749
**HDL-C, mg/dL**	43.3±14.49	41.08±11.57	0.226
**LDL-C, mg/dL**	91.64±31.69	95.45±30.36	0.405
**Triglyceride, mg/dL**	158.91±129.43	162.61±104.6	0.819
**Log hs-CRP**	0.63±0.48	0.75±0.45	0.1
**Cardiothoracic ratio, %**	0.53±0.072	0.52±0.067	0.798

*Abbreviations:* nPCR, normalized protein catabolism rate; TAC_urea_, time-averaged concentration of blood urea nitrogen; UF, ultrafiltration volume per hemodialysis period; C-calcium, corrected calcium; ALT, alanine aminotransferase; Na, serum sodium; K, serum potassium; HbA1c, glycated hemoglobin; HDL-C, high-density lipoprotein cholesterol; LDL-C, low-density lipoprotein cholesterol; log hs-CRP, logarithmic conversion of high-sensitivity C-reactive protein levels.

Besides nPCR, no significant difference was noted between basin and around basin groups.

**Table 4:** Status of protein-energy wasting and inflammation in basin and around basin groups.

Status of protein-energy wasting/inflammation	Around Basin	Basin	p value
**Normal**	67 (34.7%)	19 (30.2%)	
**High hs-CRP or low albumin**	111 (57.5%)	32 (50.8%)	
**Combined high hs-CRP and low albumin**	15 (7.8%)	12 (19.0%)	0.041

*Abbreviation:* hs-CRP, high-sensitivity C-reactive protein level.

High hs-CRP was defined as an hs-CRP level >3 mg/L.

Low albumin was defined as a serum albumin level <3.6 g/dL.

The basin group had a significantly higher proportion of combined low albumin levels and high hs-CRP levels (p = 0.041).

**Table 5:** Complications and reasons for mortality in basin and around basin groups.

	Around basin(n = 193)	Basin(n = 63)	p value
***Complications, n (%)***			
**Stroke**	16 (8.3%)	6 (9.5%)	0.797
**CAD**	12 (6.3%)	8 (12.7%)	0.108
**Cardiomegaly**	71 (36.8%)	28 (44.4%)	0.299
**Asthma**	6 (3.1%)	5 (7.9%)	0.145
**Pruritus**	42 (21.8%)	18 (28.6%)	0.305
**Hepatitis**	38 (19.7%)	13 (20.6%)	0.857
**GI bleeding**	26 (13.5%)	5 (7.9%)	0.276
**Neuropathy**	44 (22.8%)	15 (23.8%)	0.864
**Pleural effusion**	2 (1%)	1 (1.6%)	0.573
**Cirrhosis**	6 (3.1%)	2 (3.2%)	0.99
**Pul TB**	8 (4.1%)	1 (1.6%)	0.46
**Cancer**	22 (11.4%)	7 (11.1%)	0.99
**Carpal tunnel syndrome**	19 (9.8%)	4 (6.3%)	0.612
***Reason for mortality, n (%)***			
**Cardiovascular**	18 (9.3%)	12 (19%)	0.044
**Infection**	21 (10.9%)	13 (20.6%)	0.056
**Cancer**	1 (0.5%)	0	0.99
**Others**	1 (0.5%)	2 (3.2%)	0.151

Abbreviations: CAD, coronary artery disease; GI, gastrointestinal; Pul TB, pulmonary tuberculosis.

The complications of stroke and CAD were not different between basin and around basin groups. Patients living in Taipei Basin had higher 2-year cardiovascular mortality than the around Taipei Basin group (p = 0.044).

## Discussion

In this study, we could demonstrate that after adjustment for very advanced age, gender, and log hs-CRP, serum albumin levels, living in Taipei Basin, and the complications of stroke and CAD, the 2-year mortality in elderly HD patients could be predicted. In addition, patients living in Taipei Basin had a higher rate of combined protein-energy wasting and inflammation and a higher cardiovascular mortality rate, which may be the factors associated with 2-year mortality in elderly HD patients.

Due to increasing lifespan and advanced HD techniques, the prevalence of dialysis in the elderly is increasing. Several studies [1], [13], [16] have clearly identified that the severity and number of comorbid illnesses and nutritional status are risk factors associated with mortality in elderly HD patients. In a 6-month cohort observation, comorbidity score effectively predicted short-term prognosis among elderly patients starting dialysis; age was not associated with early mortality [6]. In a 12-month prospective study of dialysis patients over the age of 70 [1], mortality was significantly associated with an age of 80 years or older and peripheral vascular disease but not with diabetes, ischemic heart disease, cerebrovascular disease, chronic obstructive airways disease, sex, or treatment method. In the Dialysis Outcomes and Practice Patterns Study (DOPPS) [17], older age, catheter vascular access, albumin concentration <3.5 g/dL, phosphorus concentration <3.5 mg/dL, cancer, and congestive heart failure were all associated with 1-year mortality. In a 1-year observation, Jassal et al. [7] showed that comorbidity and the age of onset were not independent risk factors in patients aged over 65 years at the time of starting dialysis. From the above evidence, we know that age is a risk factor correlated with mortality in HD patients; however, when survival is compared within older populations, the risk of age is controversial [2], [6], [7]. It seems that the number of comorbidities and protein-energy wasting are more important for survival in elderly HD patients [6]. In the current study, the mean age was 72.64±5.64 years (ranging from 65 years to 93 years), which was close to the age defined as very advanced (75 years). Initially, in univariate logistic regression analysis, very advanced age was associated with 2-year mortality. However, upon Cox multivariate regression analysis, the effect of very advanced age disappeared. Comorbid illness as a result of stroke and CAD, low serum albumin levels, and living in Taipei Basin were correlated with 2-year mortality. These results were consistent with previous studies on the importance of comorbidity, especially that resulting from cardio- and cerebrovascular disease.

In our study population, 68 patients (26.5%) died within 2 years. Infection (50%) and cardiovascular events (44.1%) were the major causes of 2-year mortality. It is interesting that living in Taipei Basin was associated with 2-year cardiovascular mortality (OR: 2.288, 95% CI (1.03–5.06); p = 0.044) but not with 2-year infection mortality (OR: 2.13, 95% CI (0.996–4.553); p = 0.056). Evaluation of mortality in elderly dialysis patients is mostly focused on comorbidities and nutritional status. Discussion on the environment of elderly dialysis patients is limited. The rapid pace of life in the city, with lots of people and cars, can easily cause stress. The effects of dietary patterns and stress associated with urban life may increase risk factors for cerebro- and cardiovascular disease. A study on cardiovascular risk factors in urban subjects, rural subjects, and migrants showed that commonly reported cardiovascular risk factors such as systolic and diastolic blood pressure, body mass index, obesity, total cholesterol, and low-density lipoprotein were usually higher or more common in migrants than in the rural group, and usually lower or less common in migrants than in the urban group [18]. Interestingly, CRP levels were also lower in the rural group.

In our opinion, over-development of the city (with more traffic, more-production facilities, more noise, less fresh air, more crowding, and more stress) is not beneficial to human life. Comparison of living environment between city and suburb situations is complex. Air pollution is the one factor that could be quantified efficiently. Exactly, it is the matter of international concern. In northern Taiwan, analyzed data from the report of Environmental Protection Administration, the mean concentrations of PM_2.5_, NO_2_ and CO were significantly higher in Taipei Basin than that around Taipei Basin. In our knowledge, air conditions between the city and the country are different. Air pollution is usually concentrated in densely populated metropolitan areas. The 6 most common air pollutants are particulate matter (PM), lead (Pb), sulfur dioxide (SO_2_), nitrogen oxides (NO_x_), ozone (O_3_), and carbon monoxide (CO) [19]. The effects of poor air quality on human health are profound and lasting, and principally affect the respiratory and cardiovascular systems [20], [21]. Air pollution is also emerging as a risk factor for stroke [22], [23]. A recent study found an association in women between air pollution and ischemic stroke [24]. In addition, air pollution has been associated with increased incidence of and mortality from CAD [25]. In a large population and long follow-up study of 6 U.S. cities (Harvard Six Cities study), [20] significant association was noted between air pollution and mortality after adjustment for smoking, particularly in cities where pollutant levels were highest. Air pollution was also positively associated with lung cancer and cardiopulmonary disease. Extended follow-up of the Harvard Six Cities study showed that cardiovascular and lung cancer mortality were each positively associated with fine particulate air pollution (PM_2.5_) concentrations [26]. It is interesting that several studies have revealed that air pollution is associated with systemic inflammation [27]–[29]. The Taipei basin (containing the metropolitan of Taiwan –Taipei City) which is surrounded by mountains and hills has 6 million people living in it. So, the complexity of the terrain increases the difficulty of the diffusion and transfer of air pollution and we have to face more challenges on the prevention and control of air pollution. Analyzed report from the air quality status in Taiwan for the year 2009 to 2011, it is not surprised that the condition of air pollution is worse in Taipei Basin.

In our opinion, urban populations have sufficient nutritional support. However, in our study, we found that patients living in Taipei Basin had a lower nPCR than patients living around Taipei Basin. Patients living in Taipei Basin also had a higher proportion of combined protein-energy wasting and inflammation. In our knowledge, inflammation has been identified as playing a key role in the mortality of HD patients [4], [30]–[32] and the etiology and mechanism are still limited. Protein-energy wasting may worsen patient outcome by aggravating existing inflammation and heart failure and increasing susceptibility to infection. Malnutrition, inflammation, and atherosclerosis are associated with exceptionally high mortality rates. As both inflammation and inadequate nutritional intake can decrease the level of serum albumin, much of the previously reported relationship between serum albumin, malnutrition, and mortality in patients undergoing HD may be due to an inflammatory process rather than poor nutritional intake [33]–[35]. It is interesting that besides the correlation between inflammation and air pollution, in an animal study, Sun et al. investigated that long-term exposure to low concentration of PM2.5 altered vasomotor tone, induced vascular inflammation, and potentiated atherosclerosis [36]. Analyzed from two clinical trials [37], a cross-sectional study at exposure contrast of 10 µg/m^3^ PM_2.5_, carotid intima-media thickness increased by 5.9%. From the analysis of the data from the TAQMN, we found the mean concentration level of PM_2.5_ was significantly high in Taipei Basin for three consecutive years (figure 2). The studies cited above and our own observations aid explanation as to why living in Taipei Basin is a risk factor correlated with 2-year mortality in elderly HD patients.

This study has some limitations. To our knowledge, the causes of death in hemodialysis patients are generally well recognized, for example, old age, protein-energy wasting, inflammation, and cardiovascular and cerebral vascular disease. Some risk factors are yet to be clarified, but most well-known risk factors are outcomes and not the causes; some of the risk factors cannot be changed. To the best of our knowledge, comparing the advantages and disadvantages of living environments is difficult and complicated, especially with regard to elderly hemodialysis patients, who have a high prevalence of chronic comorbidities, such as cardiovascular and cerebral vascular disease. In this study, as it is closely related to our life and health, we discussed air pollution and cited associations with comorbidities of hemodialysis patients. It is important to mention that air pollution could be improved, and this fact merits the reader’s attention. Further research would be necessary to link these variables to mortality.

## Conclusion

In conclusion, this prospective observational study shows that after adjustment for protein-energy wasting and the complications of cardio- and cerebrovascular disease, living in Taipei Basin was a risk factor predicting 2-year mortality in elderly HD patients. Air pollution may be the factor that caused this phenomenon.
